# Acceptability of linking individual credit, financial, and public records data to healthcare records for suicide risk machine learning models

**DOI:** 10.1093/jamiaopen/ooae113

**Published:** 2024-10-21

**Authors:** Robert B Penfold, Hong Il Yoo, Julie E Richards, Norah L Crossnohere, Eric Johnson, Chester J Pabiniak, Anne D Renz, Nicola B Campoamor, Gregory E Simon, John F P Bridges

**Affiliations:** Kaiser Permanente Washington Health Research Institute, Seattle, WA 98101-1466, United States; Loughborough Business School, Loughborough University, Loughborough, Leicestershire LE11 3TU, United Kingdom; Kaiser Permanente Washington Health Research Institute, Seattle, WA 98101-1466, United States; College of Medicine, The Ohio State University, Columbus, OH 43210, United States; Kaiser Permanente Washington Health Research Institute, Seattle, WA 98101-1466, United States; Kaiser Permanente Washington Health Research Institute, Seattle, WA 98101-1466, United States; Kaiser Permanente Washington Health Research Institute, Seattle, WA 98101-1466, United States; College of Medicine, The Ohio State University, Columbus, OH 43210, United States; Kaiser Permanente Washington Health Research Institute, Seattle, WA 98101-1466, United States; College of Medicine, The Ohio State University, Columbus, OH 43210, United States; Bloomberg School of Public Health, Johns Hopkins University, Baltimore, MD 21205, United States

**Keywords:** suicide, data linking, privacy, discrete choice experiment

## Abstract

**Objectives:**

Individual-level information about negative life events (NLE) such as bankruptcy, foreclosure, divorce, and criminal arrest might improve the accuracy of machine learning models for suicide risk prediction. Individual-level NLE data is routinely collected by vendors such as Equifax. However, little is known about the acceptability of linking this NLE data to healthcare data. Our objective was to assess preferences for linking external NLE data to healthcare records for suicide prevention.

**Materials and Methods:**

We conducted a discrete choice experiment (DCE) among Kaiser Permanente Washington (KPWA) members. Patient partners assisted in the design and pretesting of the DCE survey. The DCE included 12 choice tasks involving 4 data linking program attributes and 3 levels within each attribute. We estimated latent class conditional logit models to derive preference weights.

**Results:**

There were 743 participants. Willingness to link data varied by type of information to be linked, demographic characteristics, and experience with NLE. Overall, 65.1% of people were willing to link data and 34.9% were more private. Trust in KPWA to safeguard data was the strongest predictor of willingness to link data.

**Discussion:**

Most respondents supported linking NLE data for suicide prevention. Contrary to expectations, People of Color and people who reported experience with NLEs were more likely to be willing to link their data.

**Conclusions:**

A majority of participants were willing to have their credit and public records data linked to healthcare records provided that conditions are in place to protect privacy and autonomy.

## Introduction

Machine learning algorithms designed to predict suicide risk can accurately identify people at high risk of making a suicide attempt^1–4^ and be used to target prevention programs such as Zero Suicide.^5^ Prediction models are already part of routine suicide prevention in health systems such as Kaiser Permanente Washington (KPWA), HealthPartners, and the Veterans Health Administration.^6^ However, about 1 in 5 suicide attempts is made by people with low predicted risk.^3^ Some of these errors may arise because the models rely exclusively on predictors from healthcare data. Adding risk factor information from data generated outside healthcare that captures negative life events (NLEs)—such as bankruptcy, foreclosure, divorce, and criminal arrest—may improve predictive accuracy by capturing circumstances that can precipitate a suicide attempt in vulnerable people. Using this information may enable earlier and more targeted suicide prevention programs.^7–9^

It is now possible to purchase NLE and other social risk factor information from vendors such as Equifax^10^ and Experian^11^ and link them, at the individual level, to healthcare data.^12^^,^^13^ However, stakeholders have expressed concerns about access to risk information (eg, privacy, data breaches, and misuse of the data to raise healthcare insurance premiums) and stigma, as well as unanticipated harms.^14^ These concerns are warranted. The exact features that make these data potentially useful to identify risk of suicide attempt are the features that raise concern—they are about things that can make people feel ashamed, isolated, and burdensome to others. We are the first to provide evidence on the preferences for linking healthcare data to social determinants of health data (and NLE data in particular) that are routinely collected outside healthcare settings.

The purpose of this study was to elicit the opinions and preferences of KPWA members regarding the linkage of external data sources to healthcare records for the purpose of improving suicide risk prediction and alerting clinicians or others regarding potential risk. We hypothesized that people may be willing to have their information linked for suicide prevention if measures are in place to protect their privacy and control over their personal information. We also hypothesized that people may be heterogeneous in their willingness to link data based on lived experiences with mental health issues and financial, gender, and legal discrimination. We further sought to provide evidence for the acceptability of using these types of data to incorporate NLEs and other social determinants of health in machine learning models more generally. This is one use case of a much larger set of questions about potentially beneficial uses of sensitive information—and how we can possibly understand and honor people’s preferences.

## Methods

### Setting

The study was conducted at KPWA, a large integrated health system serving approximately 700 000 members in Washington and Idaho. Members are generally similar to the regional population in distribution of age, sex, income, educational attainment, and race/ethnicity.^15^ Members are enrolled through a mixture of employer-sponsored insurance, individual insurance plans, Medicare, Medicaid, and other subsidized insurance programs for low-income residents. KPWA provides care through an internal group practice and a network of contracted external providers. This study was approved by the Kaiser Permanente Interregional Institutional Review Board.

### Population

A stratified random sample of 7720 members was drawn from the currently enrolled member population between October 2022 and March 2023. Males; Black, Indigenous, and people of color (BIPOC); and members with a mental health diagnosis were sampled at twice the population prevalence. Oversampling was conducted to ensure the diversity of the final sample and to ensure that the preferences of people who may regard these data as most stigmatizing or unfair were included in the study.

### Partner input

A group of 3 patient partners from the Mental Health Research Network^16^ assisted in the design and pretesting^17^ of the survey including feedback on the introductory explanation of the study, domains of concerns people have about linking data, number of questions included, appropriateness of language, and clarity of the discrete choice tasks.

### Survey design

Following partner input, we designed a discrete choice experiment (DCE). DCEs are a quantitative approach to eliciting and measuring preferences. DCE studies use survey instruments and involve presenting participants with competing products or services that have a set of attributes and levels within each attribute. Different combinations of attributes and attribute levels are presented to participants who then choose which product/service they prefer based on the combination of attributes. By repeating this choice task over many combinations of attributes, participants reveal the relative importance of attributes and attribute levels. The DCE in this study included 4 data linking program attributes and 3 levels within each attribute: type of information (financial information, legal information, and family information), specificity of suicide risk alert (specific life event prompting a suicide risk alert, area of concern only, and no detail), person notified of elevated suicide risk (Dr notified, patient notified, Dr and emergency contact notified), and permission for data use frequency (permission once, permission annually, and permission every time data are accessed).

The survey also asked a variety of questions apart from the DCE. First, we asked participants how acceptable it is to link financial, legal, and family information in the absence of any specific program attributes. There were also questions concerning respondents’ experience with mental health issues, suicide attempt(s), financial setbacks, legal problems, and family court. Questions also covered the degree to which respondents trust KPWA to protect their personal health information and financial information. Demographic data were collected at the end of the survey. Finally, respondents were asked to rate the ease of understanding and completing the survey, the degree to which their answers reflect their real preferences, and the degree to which the survey questions were relevant to them. A copy of the survey is available as online Supplementary Material.

### Experimental design

The experimental design and conduct of the DCE followed the best-practices checklist outlined by the International Society for Pharmacoeconomics and Outcomes Research.^18^^,^^19^ Ngene was used to create a balanced design with zero priors and low correlation across attribute levels.^20^ The design was D-efficient with no overlap across attribute-levels. All attributes were assumed to be categorical. The design was generated for estimating main preference effects across attributes. The design included 12 choice tasks, within standard range of common practice.^21^^,^^22^ A similar experimental design has been used successfully in other DCEs.^23–25^Figure S1 shows an example of one of the choice tasks.

### Survey administration

The survey instrument was implemented online using Illume.^26^ Potential respondents were mailed a letter describing the purpose of the survey, a description of the data that might be linked in any future application, an example DCE question, a “cheat sheet” of definitions of the attributes to be compared in the DCE (for reference while completing the survey online), contact information for the study leaders and a unique link to the online survey. Letters were sent in waves of 520 per week between October 2022 and March 2023. A random sample of 120 people in each wave was selected for telephone outreach. If reached by phone, study staff reminded participants of the letter, asked for an email, and emailed the survey link if the participant agreed. Study staff left reminder voicemails if a participant could not be reached. Participants were offered a $25 incentive to be mailed upon completing the survey.

### Statistical analysis

Choice variables were effects coded.^27–29^ The reference choices for each attribute were: family information, alerts with no details, notification of risk to Dr and emergency contact, and permission asked every time data are accessed.

We estimated latent class conditional logit models^30^^,^^31^ to derive the preference weights for each attribute level compared to the reference level. Each model assumes that individuals belong to one of *C* groups where each group has their own preferences. The output consists of each group’s preference weights, as well as membership allocation parameters that describe which group a given individual is likely to be in. Models were estimated with 4, 3, and 2 groups with the final grouping determined by the fit of the model (as measured by the Akaike Information Criterion or AIC) and intelligibility of the classes. Class membership was estimated as a function of age, gender, race, experience with mental health issues, the importance of program attributes, and trust in KPWA to protect their personal health information and financial information. All models were estimated using STATA version 17.^32^ Descriptive statistics were calculated to identify differences in sub-populations of people.

## Results

There were 743 respondents who completed or partially completed the survey (9.6% response rate). Median time to complete the survey was 15 minutes with an interquartile range between 10 and 24 minutes. There were 687 respondents who completed at least 1 discrete choice task (92.5%) and were therefore included in the DCE models. There were no significant differences in the demographic characteristics of respondents excluded from the DCE analysis.

Table 1a shows the preference weights from the latent class conditional logit model. Two classes of people were identified, which we label the Willing and the Private. The model with 2 classes had the lowest AIC and strongest face validity. The strongest driver of preferences between the 2 groups was the preference for using healthcare data only. The preference weight for healthcare data only is much larger than other weights for both groups. While the preference weight is about the same magnitude in each group, the sign is opposite; these results imply that the Private had a strong preference for using healthcare data only, whereas the Willing had a similarly strong preference for the use of expanded information. About 65% of people fall into the Willing group and 35% into the Private group. The groups were balanced with respect to demographic characteristics (see Table S1).

**Table 1.** (a) Latent class conditional logit model results.

	Willing		Private			
Proportion of respondents	65.1%		34.9%		*B_w_*-*B_p_*	
Variable	*B*	*SE*	*B*	*SE*	*z*	*P* > |*z*|
Healthcare data only	−2.089	0.092	2.585	0.102	−51.61	<.001
Financial information	−0.164	0.026	−0.361	0.088	2.12	.034
Legal information	−0.023	0.026	−0.043	0.087	0.21	.831
Family information	0.187	0.025	0.404	0.074	−2.74	.006
Alert specific reason	0.041	0.024	−0.038	0.080	0.94	.348
Alert area of concern	0.100	0.024	−0.053	0.075	1.94	.052
Alert no detail	−0.141	0.025	0.091	0.073	−3.01	.003
Notify patient	0.014	0.026	0.680	0.089	−7.12	.000
Notify Dr	0.121	0.025	0.131	0.087	−0.11	.913
Notify Dr + emerg. contact	−0.135	0.026	−0.811	0.127	5.20	.000
Permission once	−0.251	0.027	−0.298	0.087	0.51	.612
Permission annually	0.077	0.026	0.122	0.079	−0.53	.594
Permission every time	0.174	0.032	0.176	0.089	−0.02	.984

**Table:** (b) Predictors of membership in the Willing group.

Variable	*B*	*SE*	*P* > |*z*|
Trust to protect external data (high vs low)	2.121	0.221	<.001
Importance of person notified (high vs low)	−0.770	0.190	.000
Importance of permission (high vs low)	0.734	0.151	<.001
Detail change acceptability (yes vs no)	−0.306	0.133	.021
BIPOC vs Non-Hispanic White	0.285	0.213	.181
Gender (female vs male)	−0.264	0.201	.190
Mental health experience (yes vs no)	0.186	0.243	.442
Age (years)	−0.017	0.006	.009
Constant	0.451	0.651	.488

The coefficients *B* in (a) relate to the log-odds of 1 DCE choice over another. The coefficients *B* in (b) relate to the log-odds of membership in the Willing group over the Private group.

Abbreviation: DCE, discrete choice experiment.

Differences in preference weights also exist for all the attributes except frequency of permission. Respondents in both groups preferred linking family information and this was stronger in the Private group. Compared to suicide risk alerts with no detail, respondents in the Willing group preferred an alert including area of concern without naming a specific event. However, respondents in the Private group preferred alerts with no detail. With respect to who would be notified of suicide risk, respondents in the Private group preferred patient only, whereas people in the Willing group preferred Dr only.

Table 1b shows the factors associated with membership in the Willing group. There are several significant predictors; however, the strongest is whether the respondent trusts Kaiser Permanente to protect their financial, legal, and family information. People who trust Kaiser Permanente to protect these data were twice as likely to be in the Willing group compared to the Private group.

Table 2 shows the frequency that respondents endorsed experiences with issues that might influence DCE choices. A greater percentage of people in the Willing group had experience with suicide attempt(s), mental health issues, and serious financial issues. There was no difference in personal experience related to serious legal issues or family court.

**Table 2.** Have you had personal experience with one of these issues or been personally impacted by someone who did?

	Willing		Private		Overall		
	*n*	%	*n*	%	*n*	%	*P* Chi sq.
Suicide attempt	239	49.3	95	36.8	334	45.0	<.001
Mental health issue	385	79.4	149	57.8	534	71.9	<.001
Serious financial issue	241	49.7	93	36.0	334	45.6	<.001
Serious legal issue	125	25.8	65	25.2	190	25.6	.851
Family court	130	26.8	59	22.9	189	25.4	.241

Table S2 further shows differences in the acceptability of linking legal (eg, criminal arrest) and family court data between BIPOC respondents and non-BIPOC respondents. About 17% of BIPOC respondents felt linking legal data was unacceptable; however, about 26% of non-BIPOC respondents found linking such data unacceptable (*P* = .032). The difference was stronger for family court data with 9.9% of BIPOC respondents reporting linking such data unacceptable compared to 19.3% of non-BIPOC respondents (*P* = .006).

Table 3 reports the frequency with which respondents found it acceptable, potentially acceptable, or not acceptable to link data outside healthcare in the absence of any specific program attributes. Of all respondents, 67.4% found it acceptable or potentially acceptable to link financial data. Similarly, 76.6% of people found it acceptable or potentially acceptable to link legal information and 81.6% family information. However, the proportion of people in the Willing and Private groups who endorsed acceptability was significantly different across all information types.

**Table 3.** Acceptability of linking data by type—naïve to privacy controls. For you personally, how acceptable is it to use this information for suicide risk prediction?

	Financial information^a^				Legal information^a^				Family information^a^			
	Willing		Private		Willing		Private		Willing		Private	
Acceptability	*n*	%	*n*	%	*n*	%	*n*	%	*n*	%	*n*	%
Acceptable	128	26.5	25	9.7	203	41.9	56	22.0	282	58.6	70	28.2
Potentially acceptable	255	52.7	93	36.2	214	44.1	96	37.7	162	33.7	92	37.1
Not acceptable	101	20.8	139	54.1	68	14.0	103	40.4	37	7.7	86	34.7
Missing	1	–	1	–	–	–	3	–	4	–	10	–
Total	485		258		485		258		485		258	

a *P* Chi sq. < .001.

We conducted sensitivity analyses where willingness to have financial, legal, and family information linked is nested within NLE experience. The pattern of acceptability when comparing the Willing and the Private does not change by NLE experience group. Generally, people in the Willing group are more likely to be willing to link their data if they have experience with a particular NLE (see Tables S4-S6).

Table 4 shows the frequency with which respondents endorsed their trust in KPWA to protect their personal health information and their financial, legal, and family information. Overall, 86.8% (*n* = 645) of respondents reported that they at least somewhat trust KPWA to protect their personal health information and 73.3% (*n* = 545) at least somewhat trust KPWA to protect their financial, legal, and family information. However, trust diverged greatly between the 2 groups. More than 80% (*n* = 390) of people in the Willing group reported trusting KPWA a fair amount or a great deal to protect their personal health information whereas only 25% (*n* = 48) of people in the Private group reported this level of trust. Even more starkly, about 68% (*n* = 329) of people in the Willing group reported trusting KPWA a fair amount or a great deal to protect their financial, legal, and family information compared to only 3.2% (*n* = 6) of people in Private group.

**Table 4.** Trust in Kaiser Permanente to protect personal information. How much, if at all, do you trust Kaiser Permanente to protect your information?

	Personal health information^a^				Financial, legal, and family information^a^					
	Willing		Private		Willing		Private		Overall	
Trust KP	*n*	%	*n*	%	*n*	%	*n*	%	*n*	%
A great deal	155	32.0	12	6.3	125	25.8	5	2.7	130	19.4
A fair amount	235	48.4	36	19.0	204	42.1	1	0.5	205	30.6
Somewhat	92	19.0	56	29.5	153	31.5	3	1.6	156	23.3
Not much	3	0.6	68	35.7	3	0.6	112	60.6	115	17.2
Not at all	0	0.0	18	9.5	0	0.0	64	34.6	64	9.6
Missing	0	–	68	–	0	–	73	–	73	–
Total	485	65.3	258	34.7	485	65.3	258	34.7	743	100

a *P* Chi sq. < .001.

A final measure of the acceptability of any data linking program is the proportion of people who chose a combination of attributes other than “healthcare data only” as being the most preferred for at least 1 of the 12 choice tasks. Overall, 84% (*n* = 624) of people selected at least 1 combination of data-linking attributes as the most preferred. However, this differed between the Willing (91.8%) and Private (69.4%).

## Discussion

Machine learning and the availability of data regarding NLEs may make it possible to have greater predictive power in health systems’ efforts to target suicide prevention. While there has been reluctance across stakeholders to move in this direction (both for research and practice), the evidence from this sample indicates that linking credit and public records data to healthcare data for the purposes of suicide risk prediction is socially acceptable. Most people who responded to this survey support linking data for this purpose provided certain conditions are in place to protect privacy and autonomy; however, a little more than 1/3 of people prefer that only healthcare data be used for risk prediction.

A second important finding is that people who have experience with mental health issues, suicide, and significant financial setbacks were *more* likely to be willing to link their data; not less. We hypothesized that stigma associated with these experiences might make people less willing to link data. It appears that people with these experiences understand how these data might improve risk prediction. Similarly, BIPOC respondents were more likely to support linking legal and family court data for suicide risk prediction. We hypothesized that BIPOC respondents would regard these data as biased and therefore be less willing to link these data because BIPOC individuals have often been treated unfairly by the courts. It is possible that BIPOC respondents recognize that biased treatment by the court system could be a significant predictor of suicide risk.

Looking at the results of the DCE, it appears that the following expanded data program might be sufficiently acceptable to most people if it were to be operationalized:

- Link all the financial, legal, and family data available to maximize prediction accuracy.
- Communicate any notification of increased risk to the person’s doctor only to maximize privacy.
- Only alert the doctor to an area of concern. For example, “financial stress” rather than “bankruptcy.” This allows the person to divulge whatever level of detail they choose when their clinician opens a conversation.
- Obtain permission to access and link records every year at open enrollment and make it easy for people to revoke their consent. This gives people control over their information.

In the introduction to the survey (see Supplementary Materials for the full survey), we also specified some rules that we believe would need to be mandatory.

- No information about a person’s healthcare would be released to credit agencies or public records or any other entity.
- Using financial information would have no impact on credit scores and would not generate a soft or hard “pull” on a person’s credit.
- Financial and public records data would never be used to sell people insurance products, increase insurance premiums, limit insurance coverage, or limit healthcare provided to anyone in any way.

Another consideration that the survey did not ask about directly, but many respondents communicated in open-ended questions, is that the acceptability of linking data is contingent on the degree of improvement in risk prediction. Many respondents were skeptical that these data could improve suicide risk prediction and that their opinion would be more favorable if they knew using these data would lead to fewer suicide attempts. We plan to do this research. Questions about operationalization and clinical workflow are moot if the data do not improve risk prediction.

Thinking about operationalization, the nature of communication from clinicians and care teams about suicide risk would need to be cautious. We envision improving the accuracy of machine learning models to correctly predict not only who is at high risk of making a suicide attempt but also when vulnerable people are at increased risk proximate to a NLE—similar to proposals that would use social media data to identify suicide risk.^33^ Newly developed models would augment existing efforts that use machine learning to identify and engage at-risk people in suicide prevention.^34^^,^^35^ People identified as high risk would receive personalized outreach to check in on their well-being or additional attention at the point-of-care. Importantly, this approach would not focus on imminent risk of suicide. The vast majority of people who experience bankruptcy, divorce, foreclosure, and other major NLEs do not attempt suicide. Thus, the nature of communication would be a non-demanding expression of care or “caring contact”^36^ where a clinician expresses interest in a person’s wellbeing and opens a conversation about what, if anything, might be happening in their life that they want to talk about. Caring contacts have been demonstrated to reduce suicide attempts.^36–38^

Developing and maintaining trust would be a critical component for successfully linking credit and public records data to healthcare data in a suicide prevention program. This includes trust in the health system to protect people’s data and in clinicians to use the information in ways that maintain privacy and autonomy. This would require developing transparency around data acquisition and use, clearly communicating information security policies, and giving people regular opportunities to revoke their permission to acquire and use the data. It will be critical to use the results of this study and prior qualitative studies^39–41^ to implement newly improved models in a way that protects people from potential harms and maintain trust.

Several limitations should be noted. First, we oversampled KPWA members with documented mental health diagnoses and BIPOC members. It is advisable to oversample if there are hypothesized latent classes in the event that minority preferences belong to a specific latent class.^27^ When conducting latent class analyses, diversity of sample is preferred over representation. We reasoned that some individuals would be more influenced by stigma and/or unfair treatment by the systems generating NLE data outside healthcare. Thus, while the demographic composition of the sample does not mirror the US population, oversampling allowed us to evaluate the preferences of BIPOC individuals with sufficient statistical power and to conduct latent class analyses. Moreover, any health system contemplating the use of these types of data would want to consider how the study population is similar to that health system’s population, not to the US population. However, it is true that generalizability of our findings to the US population as a whole is unknown. We look forward to replicating the experiment in other health systems as recommended by others^42^ and sharing the instrument for others to do so as well.

Second, the survey response rate was low compared to some publication guidelines. However, our response rate was comparable to other DCEs with response rates around 8%-10%.^43–45^ In DCE studies with higher response rates (18.1%-24.1%), participants were offered unusually high incentives ($60-$100 US).^46^

Third, there was perhaps a missed opportunity to ask respondents directly about other factors that may have influenced their preferences toward linking information, including digital literacy, financial literacy, and education level. Familiarity with data security and privacy measures could certainly impact preferences, though, previous studies that have examined the relationship between health literacy and preferences report a high correlation in preferences between those with high and low health literacy.^47^ We did ask respondents about their experience completing the survey and most respondents indicated the survey was easy to understand, easy to complete, that their answers reflected their true preferences, and that the questions were relevant to them (see Table S9). There was not a statistically significant difference between the Willing and the Private with respect to ease of understanding or ease of answering. Respondents in the Private group were more likely to strongly agree that their answers reflect their real preferences. Respondents in the Private group were also more likely to strongly agree that the questions were relevant to them.

We also conducted sensitivity analyses to test whether people with NLE experience reported different experiences vis-à-vis completing the survey (ie, to test the potential that digital literacy was a confounder). We conducted Chi-square analyses to measure the difference of proportions between the survey experience questions and willingness to link data but limited to people who endorsed experience within each NLE, mental health issues, and suicidality (see Tables S10-S13). Again, there was no significant difference in proportions except for the relevance question, where people endorsing experience with mental health issues were more likely to report that the questions were relevant.

Fourth, we conducted this survey with respect to a specific use case, where the risk of death may make respondents more willing to link information. It is almost certain that people have different willingness to link data for a life-or-death use case. It would not be surprising that people are unwilling to have their data linked for lower risk outcomes or use cases. Further work is needed to address preferences for data linking across the full spectrum of potential uses. If acceptable, health systems would likely use these data for outcomes such as psychiatric hospitalization, emergency department visits, and for identifying people with social needs more generally.

In this study, we demonstrate the social acceptability of expanding data sources to include credit bureau and public records that improve the capture of NLEs and other social determinants of health and may increase our ability to target suicide prevention strategies in health systems. We also demonstrate that this would be consistent with health system members’ preferences. Our findings demonstrate the acceptability of conducting research regarding how much these data might improve risk prediction in real-world conditions. If successful, extensive patient, clinician, and health system engagement will be vital to supporting successful implementation of new models in a way that respects people’s privacy and autonomy.

## Supplementary Material

ooae113_Supplementary_Data

## Data Availability

Data are available from the National Data Archive through the National Institute of Mental Health.
