# Supplementary material for: Acceptability of linking individual credit, financial, and public records data to healthcare records for suicide risk machine learning models
Source: JAMIA Open. 2024 Oct 21;7(4):ooae113. doi: 10.1093/jamiaopen/ooae113 (PMC11493183; doi:10.1093/jamiaopen/ooae113)
Supplement: ooae113_Supplementary_Data [file ooae113_supplementary_data.docx]

**Supplementary Information**

**Acceptability of Linking Individual Credit, Financial and Public Records Data to Healthcare Records for Suicide Risk Machine Learning Models**

Robert B. Penfold; Hong Il Yoo; Julie E. Richards; Norah L. Crossnohere; Nicola B. Campoamor; Eric Johnson; Chester J. Pabiniak; Anne D. Renz; Nirmala D. Rupan; Gregory E. Simon; John F.P. Bridges

Corresponding author: robert.b.penfold@kp.org

**The PDF file includes:**

Materials and Methods

Figs. S1 to S2

Tables S1 to S14

Materials and Methods

Full Survey Instrument

**Introduction**

We are constantly looking at ways to improve member care and we need your help. We are a team of researchers at Kaiser Permanente working to collect member preferences about using financial and public records information for suicide prevention. As part of the Preferences for Data Use study, we would like to get your thoughts on the following survey. As a thank you for sharing your thoughts, we will send you $25 in the mail.

We want to know what members think about using credit and financial information (like credit score, debt-to-income ratio, and recent changes in spending levels) and public records information (like records of divorce, bankruptcy, and arrests) to help identify people at risk of making a suicide attempt. Even if you have no familiarity with suicide, your opinions are important to us.

[page break]

Kaiser Permanente Washington already uses information from patients’ medical records to identify people who might be at higher risk of making a suicide attempt. That information alerts our mental health therapists and doctors to when they should ask additional questions about suicide.

Information in medical records, such as a mental health diagnosis, can only tell us so much about suicide risk. Information about stressful events that are recorded outside of health care, such as divorce, credit problems, foreclosure, bankruptcy, and arrests may help us identify people we might miss from medical record information.

No single piece of information can accurately identify people at risk but combining financial or public records information with medical records information could be used in the future for improving suicide risk prediction.

[page break]

Any use of credit, financial or public records data by Kaiser Permanente would have to follow some specific rules:

- No information from Kaiser Permanente about your health care would go back to credit agencies or public records.
- Using financial information would have no impact on credit scores and would not generate a soft or hard “pull” on your credit.
- Financial and public records data would never be used to sell you insurance products, increase insurance premiums, limit insurance coverage, or limit health care provided to anyone in any way.

[page break]

We want to know what members think about:

1. What kind of credit, financial and public records information should be used?
2. How much specific detail should be included?
3. Who should have access to that information?
4. How should people be asked about permission to use that information?
5. How should health care teams respond to information that someone might be at higher risk?

[page break]

We will ask what you think about these questions by asking you to compare different types of information that could be used in combination with different levels of privacy protection and tell us which you prefer. Each choice has a different combination of:

- Type of information used
- Detail of information
- Who would be notified about suicide risk
- How often we ask your permission to use your information.

This will help us understand how comfortable members are with the use of this data, who should have access to it and what to do with it.

[page break]

In the comparison tasks that we will ask you to do, we use some short labels to make the comparisons easier. The labels are defined below. You can refer to the letter or email we sent for these definitions if you need to.

Each of the comparisons has 4 factors and 3 options within each of them:

Information that would be made available

Our program could involve combining different types of information recorded outside of health care records. We will ask you to compare programs that include these three different types of information:

- **Financial**: we would access financial records information such as credit score, debt-to-income ratio, and recent changes in spending levels

For you personally, how acceptable is it to use financial information for suicide risk prediction?

Acceptable

Potentially acceptable

Not acceptable

- **Legal**: we would access public records such as traffic tickets, arrests, and criminal convictions

For you personally, how acceptable is it to use legal information for suicide risk prediction?

Acceptable

Potentially acceptable

- Not acceptable

  **Family**: we would access public records such as marriage, separation, divorce, and child custody

For you personally, how acceptable is it to use family information for suicide risk prediction?

Acceptable

Potentially acceptable

Not acceptable

For you personally, how important is the type of information included?

1. Important

2. Somewhat important

3. Not important

[page break]

Level of detail of the information shared

Suicide risk prediction could involve different levels of detail when doctors or members are alerted to increased suicide risk. We will ask you to compare programs that include these three different degrees of specificity:

- **Alert, particular reason**: The alert indicates a specific stressful event occurred – such as divorce, bankruptcy or arrest
- **Alert, area of concern**: The alert indicates some stressful event occurred, but not the details
- **Alert, no details**: The alert contains no information about why an alert occurred – your doctor only knows that you may be at higher risk of a suicide attempt

For you personally, does the level of detail shared change how acceptable is it to use this information for suicide risk prediction?

Yes

Somewhat

No

For you personally, how important is the level of detail of information in the alert?

Important

Somewhat important

Not important

[page break]

Person notified

Our program could involve different people being notified. We will ask you to compare programs that include these three different types of notification:

- **Dr. notification**: Your doctor is the only one alerted and reaches out to you
- **Dr. & emergency contact notification**: Your doctor and your emergency contact are alerted
- **Patient notification**: Only you receive an alert asking you to contact your doctor

For you personally, does the person notified change how acceptable is it to use this information for suicide risk prediction?

Yes

Somewhat

No

For you personally, how important is the person or people notified?

Important

Somewhat important

Not important

[page break]

How often we would ask for your permission

Our program could involve different ways of asking your permission to view this information. We will ask you to compare programs that include a rule for how often we ask for your permission

- **Only once**: We ask your permission to use this information the first time only
- **Once per year**: We ask your permission to use this information every year during open enrollment
- **Every time**: We ask your permission every time we want to use your information

For you personally, does how often we ask your permission change how acceptable is it to use this information for suicide risk prediction?

Yes

Somewhat

No

For you personally, how important is the frequency of asking your permission?

Important

Somewhat important

Not important

[page break]

Before health systems like Kaiser Permanente used financial, credit, legal and family information, researchers would like to find out how much that information actually helps to identify people at high risk. To do that, they would connect financial, credit, legal and family information with health records – but remove names and any other identifying information. How acceptable is that?

Acceptable

Neutral

Not acceptable

[page break]

Before we continue with the rest of the survey, is there anything you would like us to know about your thoughts or experiences on personal data use generally or use of specific types of information?

[ Free text box with 1500 character limit ]

[page break]

**Next, is an example of the types of comparisons we will ask you to make.**

A person like you, who we will call Sam, had to choose between the two sets of rules for data use below. Sam considers the first set of rules, which involves combining financial information with health care information. An alert about this general area of concern would be generated (but not the details) and both the doctor and emergency contact would be notified. Sam would only be asked for permission to access these data once.

Sam then considers the second set of rules. The program involves combining legal information with health care information. An alert regarding the specific details of the legal problem would occur (for example, arrested for driving under the influence). That alert would involve a message only to Sam asking Sam to make an appointment to discuss how this particular event might be affecting Sam. Sam would be asked for permission every time Kaiser Permanente accessed Sam’s public legal records.

Sam compares the programs and feels most comfortable with the characteristics of the second program.

| **Characteristic** | **Expanded Info 1** | **Expanded Info 2** | **Health care Info Only** |
| --- | --- | --- | --- |
| Information that would be made available | Financial | Legal | Kaiser Permanente should not access information outside health care under either of these sets of rules |
| Details of the information shared | Alert, area of concern | Alert, particular reason |  |
| Person notified | Dr. & emergency contact | Patient alert |  |
| How often we would ask for your permission | Only once | Every time |  |
| Which do you prefer? | □ | □ | □ |

[page break]

Now it is your turn to consider different types of information and rules for using it. In this survey you will see 14 different pairs of choices. In each pair, you will be asked which one you would choose if both data use programs were real and available to you. You can also choose “health care information only” which means you prefer neither expanded information program for suicide risk prediction.

Please remember to consider all the factors of each set of choices.**We really appreciate your time and look forward to learning more about your opinions.**

If you have questions about how to complete the rest of the survey, you can call the KPWHRI Survey Research Program for assistance at 1-XXX-XXX-XXXX. You can leave a voicemail with your name, number, and a good time to reach you.

[page break]

Select the box under the program that you would choose.

**Variable name: Task1** *(not shown to participants)*

|  | | |  |
| --- | --- | --- | --- |
| **Characteristics** | **Expanded Info 1** | **Expanded Info 2** | **Health care Info Only** |
| Information that would be made available | Legal | Family | Kaiser Permanente should not access information outside health care under either of these sets of rules |
| Details of the information shared | Alert, area of concern | Alert, particular reason |  |
| Person notified | Patient alert | Dr. & emergency contact |  |
| How often we would ask for your permission | Every time | Once per year |  |
| Which would you prefer? | **☐** | **☐** | **☐** |

[page break]

**Variable name: Task2** *(not shown to participants)*

Select the box under the program that you would choose.

|  | | |  |
| --- | --- | --- | --- |
| **Characteristics** | **Expanded Info 1** | **Expanded Info 2** | **Health care Info Only** |
| Information that would be made available | Family | Legal | Kaiser Permanente should not access information outside healthcare under either of these sets of rules |
| Details of the information shared | Alert, particular reason | Alert, no details |  |
| Person notified | Patient alert | Dr. & emergency contact |  |
| How often we would ask for your permission | Every time | Only once |  |
| Which would you prefer? | **☐** | **☐** | **☐** |

[page break]

**Variable name: Task3** *(not shown to participants)*

Select the box under the program that you would choose.

|  | | |  |
| --- | --- | --- | --- |
| **Characteristics** | **Expanded Info 1** | **Expanded Info 2** | **Health care Info Only** |
| Information that would be made available | Legal | Financial | Kaiser Permanente should not access information outside health care under either of these sets of rules |
| Details of the information shared | Alert, particular reason | Alert, area of concern |  |
| Person notified | Dr. & emergency contact | Dr. alert |  |
| How often we would ask for your permission | Every time | Once per year |  |
| Which would you prefer? | **☐** | **☐** | **☐** |

[page break]

**Variable name: Task4** *(not shown to participants)*

Select the box under the program that you would choose.

|  | | |  |
| --- | --- | --- | --- |
| **Characteristics** | **Expanded Info 1** | **Expanded Info 2** | **Health care Info Only** |
| Information that would be made available | Family | Financial | Kaiser Permanente should not access information outside health care under either of these sets of rules |
| Details of the information shared | Alert, no details | Alert, area of concern |  |
| Person notified | Patient alert | Dr. alert |  |
| How often we would ask for your permission | Only once | Every time |  |
| Which would you prefer? | **☐** | **☐** | **☐** |

[page break]

**Variable name: Task5** *(not shown to participants)*

Select the box under the program that you would choose.

|  | | |  |
| --- | --- | --- | --- |
| **Characteristics** | **Expanded Info 1** | **Expanded Info 2** | **Health care Info Only** |
| Information that would be made available | Legal | Financial | Kaiser Permanente should not access information outside health care under either of these sets of rules |
| Details of the information shared | Alert, area of concern | Alert, particular reason |  |
| Person notified | Dr. & emergency contact | Patient alert |  |
| How often we would ask for your permission | Once per year | Only once |  |
| Which would you prefer? | **☐** | **☐** | **☐** |
| [page break]  **Variable name: Task6** *(not shown to participants)*  Select the box under the program that you would choose. | | |  |
| **Characteristics** | **Expanded Info 1** | **Expanded Info 2** | **Health care Info Only** |
| Information that would be made available | Family | Legal | Kaiser Permanente should not access information outside health care under either of these sets of rules |
| Details of the information shared | Alert, no details | Alert, particular reason |  |
| Person notified | Dr. alert | Dr. & emergency contact |  |
| How often we would ask for your permission | Once per year | Only once |  |
| Which would you prefer? | **☐** | **☐** | **☐** |

[page break]

| **Variable name: Task7** *(not shown to participants)*  Select the box under the program that you would choose. | | |  |
| --- | --- | --- | --- |
| **Characteristics** | **Expanded Info 1** | **Expanded Info 2** | **Health care Info Only** |
| Information that would be made available | Financial | Family | Kaiser Permanente should not access information outside health care under either of these sets of rules |
| Details of the information shared | Alert, area of concern | Alert, no details |  |
| Person notified | Patient alert | Dr. alert |  |
| How often we would ask for your permission | Only once | Every time |  |
| Which would you prefer? | **☐** | **☐** | **☐** |

[page break]

| **Variable name: Task8** *(not shown to participants)*  Select the box under the program that you would choose. | | |  |
| --- | --- | --- | --- |
| **Characteristics** | **Expanded Info 1** | **Expanded Info 2** | **Health care Info Only** |
| Information that would be made available | Financial | Legal | Kaiser Permanente should not access information outside health care under either of these sets of rules |
| Details of the information shared | Alert, no details | Alert, particular reason |  |
| Person notified | Dr. & emergency contact | Dr. alert |  |
| How often we would ask for your permission | Every time | Every time |  |
| Which would you prefer? | **☐** | **☐** | **☐** |

[page break]

| **Variable name: Task9** *(not shown to participants)*  Select the box under the program that you would choose. | | |  |
| --- | --- | --- | --- |
| **Characteristics** | **Expanded Info 1** | **Expanded Info 2** | **Health care Info Only** |
| Information that would be made available | Family | Financial | Kaiser Permanente should not access information outside health care under either of these sets of rules |
| Details of the information shared | Alert, area of concern | Alert, no details |  |
| Person notified | Dr. alert | Patient alert |  |
| How often we would ask for your permission | Only once | Once per year |  |
| Which would you prefer? | **☐** | **☐** | **☐** |

[page break]

| **Variable name: Task10** *(not shown to participants)*  Select the box under the program that you would choose. | | |  |
| --- | --- | --- | --- |
| **Characteristics** | **Expanded Info 1** | **Expanded Info 2** | **Health care Info Only** |
| Information that would be made available | Financial | Legal | Kaiser Permanente should not access information outside health care under either of these sets of rules |
| Details of the information shared | Alert, particular reason | Alert, no details |  |
| Person notified | Dr. & emergency contact | Patient alert |  |
| How often we would ask for your permission | Once per year | Every time |  |
| Which would you prefer? | **☐** | **☐** | **☐** |

page break]

**Variable name: Task11** *(not shown to participants)*

| Select the box under the program that you would choose. | | |  |
| --- | --- | --- | --- |
| **Characteristics** | **Expanded Info 1** | **Expanded Info 2** | **Health care Info Only** |
| Information that would be made available | Financial | Family | Kaiser Permanente should not access information outside health care under either of these sets of rules |
| Details of the information shared | Alert, no details | Alert, area of concern |  |
| Person notified | Dr. alert | Patient alert |  |
| How often we would ask for your permission | Only once | Once per year |  |
| Which would you prefer? | **☐** | **☐** | **☐** |

[page break]

| **Variable name: Task12** *(not shown to participants)*  Select the box under the program that you would choose. | | |  |
| --- | --- | --- | --- |
| **Characteristics** | **Expanded Info 1** | **Expanded Info 2** | **Health care Info Only** |
| Information that would be made available | Legal | Family | Kaiser Permanente should not access information outside health care under either of these sets of rules |
| Details of the information shared | Alert, particular reason | Alert, area of concern |  |
| Person notified | Dr. alert | Dr. & emergency contact |  |
| How often we would ask for your permission | Once per year | Only once |  |
| Which would you prefer? | **☐** | **☐** | **☐** |

[page break]

**Variable name: Task13** *(not shown to participants)*

| Select the box under the program that you would choose. | | |  |
| --- | --- | --- | --- |
| **Characteristics** | **Expanded Info 1** | **Expanded Info 2** | **Health care Info Only** |
| Information that would be made available | Family | Financial | Kaiser Permanente should not access information outside health care under either of these sets of rules |
| Details of the information shared | Alert, no details | Alert, particular reason |  |
| Person notified | Patient alert | Dr. alert |  |
| How often we would ask for your permission | Every time | Only once |  |
| Which would you prefer? | **☐** | **☐** | **☐** |

[page break]

**Variable name: Task14** *(not shown to participants)*

Select the box under the program that you would choose.

|  |  |  |  |
| --- | --- | --- | --- |
| **Characteristics** | **Expanded Info 1** | **Expanded Info 2** | **Health care Info Only** |
| Information that would be made available | Legal | Financial | Kaiser Permanente should not access information outside health care under either of these sets of rules |
| Details of the information shared | Alert, particular reason | Alert, area of concern |  |
| Person notified | Dr. & emergency contact | Dr. alert |  |
| How often we would ask for your permission | Every time | Once per year |  |
| Which would you prefer? | **☐** | **☐** | **☐** |

[page break]

Thank you for sharing your preferences. We would like to know about your experience comparing the data use programs that you just completed. Please mark your answers in the grid.

|  | Strongly disagree | Disagree | Neither | Agree | Strongly agree |
| --- | --- | --- | --- | --- | --- |
| The questions were easy to understand | **☐** | **☐** | **☐** | **☐** | **☐** |
| The questions were easy to answer | **☐** | **☐** | **☐** | **☐** | **☐** |
| My answers showed my real preferences | **☐** | **☐** | **☐** | **☐** | **☐** |
| The questions were relevant to me | **☐** | **☐** | **☐** | **☐** | **☐** |

Please share any comments you may have about your experience comparing the data use programs

[Free text box, 1500 character limit]

[page break]

**Attempt.** Thank you for sharing your experience. Please tell us a little bit more about you.

Have you had personal experience with suicide attempt or been personally impacted by someone who did?

- 1. Yes
  2. Not that I know of

[LOGIC: If yes then display:]

If you are having thoughts about self-harm, please contact any of the following resources.

- 1. These two resources are available during business hours:
     1. **Your Kaiser Permanente primary care clinic**. Please call the clinic, because secure messages may not be read for a day or two.
     2. **Kaiser Permanente’s Mental Health and Wellness department**: (888) xxx-xxxx
  2. These two resources are available 24 hours a day:
     1. **National Suicide Prevention Lifeline**: 988
     2. **Kaiser Permanente’s Consulting Nurse**: (800) xxx-xxxx

[page break]

1. **Mental Health.** Have you had personal experience with mental health issues or been personally impacted by someone who did?
   1. Yes
   2. Not that I know of

[page break]

1. **Financial.** Have you had personal experience with serious financial setbacks or been personally impacted by someone who did?
   1. Yes
   2. Not that I know of

[page break]

1. **Legal.** Have you had personal experience with serious legal issues or been personally impacted by someone who did?
   1. Yes
   2. Not that I know of

[page break]

1. **Family_court.** Have you had personal experience with family court or been personally impacted by someone who did?
   1. Yes
   2. Not that I know of

[page break]

1. **TRUST_KP.** How much, if at all, do you trust Kaiser Permanente to protect your personal health information?
   1. A great deal
   2. A fair amount
   3. Somewhat
   4. Not much
   5. Not at all
2. **TRUST_KP.** How much, if at all, do you trust Kaiser Permanente to protect your credit, financial, and legal information?
   1. A great deal
   2. A fair amount
   3. Somewhat
   4. Not much
   5. Not at all

[page break]

1. **COMMENTS.** Anything else you would like to tell us?

Free text box [3000 character limit]

[page break]

1. Demographics

**AGE** What is your age? TEXT BOX, 18 – 89

[page break]

**SEX** What sex were you assigned at birth on your original birth certificate?

1. Male
2. Female

[page break]

**GENDER** What is your current gender?

1. Male
2. Female
3. Transgender
4. I use a different term: [free text box]

[page break]

**ETHNICITY** What is your ethnicity?

1 Hispanic or Latino

2 Not Hispanic or Latino

[page break]

**RACE** What is your race? – (Please check all that apply)

White/Caucasian **RACE_WHITE**

Black or African-American **RACE_AA**

Asian **RACE_ASIAN**

Pacific Islander **RACE_PAC_ISL**

American Indian or Alaska Native **RACE_NATIVE**

Other       **RACE_OTHER_SVY ;   RACE_OTHER_TEXT**

[page break]

**ENDADDR**

Thank you for sharing your thoughts.  **We really appreciate your help!**

We will mail your $25 within the next few weeks. Where should we send it?

(SHOW ADDRESS IN RECORD.)

1. ADDRESS IS THE SAME (SKP TO STUDYTHANKS)
2. UPDATE ADDRESS (SHOW FIELDS FOR UPDATING)

[page break]

**END_PAGE** If you have concerns about this topic or questions asked in the survey, Dr. Penfold will be happy to talk with you. You can reach him at 206-xxx-xxxx or [robert.b.penfold@kp.org](mailto:robert.b.penfold@kp.org).

Thanks again.

Fig. S1.

|  | | |  |
| --- | --- | --- | --- |
| **Characteristics** | **Expanded Program 1** | **Expanded Program 2** | **Healthcare Data Only** |
| Information that would be made available | Legal | Family | Kaiser Permanente should not access information outside healthcare under either of these programs |
| Specificity of the information shared | Alert, area of concern | Alert, particular reason |  |
| Person notified | Patient alert | Dr. & emergency contact |  |
| How often we would ask for your permission | Every time | Once per year |  |
| Which would you prefer? | **☐** | **☐** | **☐** |

An example discrete choice experiment question.

Expanded Program 1 and Expanded Program 2 include combinations of the levels of program attributes. The healthcare data only choice was also presented as an opt-out alternative for every choice task. Respondents were asked to choose which one of these 3 programs they most prefer.

Fig. S2.

* Excludes the alternative specific constant (opt-out) “healthcare data only”

Table S1. Self-reported demographic characteristics

|  | Willing | | Private | | Overall | |  |
| --- | --- | --- | --- | --- | --- | --- | --- |
|  | **n** | **%** | **n** | **%** | **n** | **%** | Pr Chisq |
| Age |  |  |  |  |  |  | 0.473 |
| Generation Z (1997-2010) | 45 | 9.3 | 9 | 5.1 | 54 | 8.1 |  |
| Millenial (1981-1996) | 149 | 30.7 | 58 | 32.6 | 207 | 31.2 |  |
| Generation X (1965-1980) | 134 | 27.6 | 54 | 30.3 | 188 | 28.4 |  |
| Baby Boomer (1946-1964) | 143 | 29.5 | 53 | 29.8 | 196 | 29.6 |  |
| Silent Generation (1928-1945) | 14 | 2.9 | 4 | 2.3 | 18 | 2.7 |  |
| Missing | - | - | 80 | - | 80 | 10.8 |  |
| Sex at Birth |  |  |  |  |  |  | 0.94 |
| Male | 231 | 47.6 | 88 | 47.3 | 319 | 47.5 |  |
| Female | 254 | 52.4 | 98 | 52.7 | 352 | 52.5 |  |
| Missing | - | - | 72 | - | 72 | 9.7 |  |
| Race |  |  |  |  |  |  | 0.11 |
| White | 305 | 63.4 | 123 | 69.1 | 428 | 57.6 |  |
| Black/African American | 30 | 6.2 | 7 | 3.9 | 37 | 5.0 |  |
| Asian | 84 | 17.5 | 26 | 14.6 | 110 | 14.8 |  |
| Pacific Islander / Alaska Native | 10 | 2.1 | 1 | 0.6 | 11 | 1.5 |  |
| Native American | 1 | 0.2 | 3 | 1.7 | 4 | 0.5 |  |
| Other | 5 | 1 | 1 | 0.6 | 6 | 0.8 |  |
| White + other | 40 | 8.32 | 17 | 9.6 | 57 | 7.7 |  |
| Hispanic/Latino | 6 | 1.3 | 0 | 0.0 | 6 | 0.8 |  |
| BIPOC | 178 | 36.7 | 58 | 31.0 | 236 | 31.8 |  |
| Missing | 4 | - | 80 | - | 84 | 11.3 |  |
| Ethnicity |  |  |  |  |  |  | 0.337 |
| Hispanic/Latino | 40 | 8.3 | 11 | 6.0 | 51 | 7.7 |  |
| Total | 485 | 65.3 | 258 | 34.7 | 743 | 100 |  |

Sample Demographics

Table S2.

|  | | | | | | | |
| --- | --- | --- | --- | --- | --- | --- | --- |
|  |  |  | **BIPOC** |  |  |  |  |
|  | No | | Yes | | Total | |  |
|  | n | % | n | % | n | % | Pr > Chisq |
| Acceptable | 150 | 34.4 | 89 | 37.9 | 239 | 32.2 |  |
| Potentially acceptable | 173 | 39.7 | 106 | 45.1 | 279 | 37.6 |  |
| Not acceptable | 113 | 25.9 | 40 | 17.0 | 153 | 20.6 |  |
| Total | 436 | 100.0 | 235 | 100.0 | 671 | 90.3 | 0.032 |

Differences in acceptability of using legal data by BIPOC status.

Question: For you personally, how acceptable is it to use legal information for suicide risk prediction?

Table S3.

|  | | | | | | | |
| --- | --- | --- | --- | --- | --- | --- | --- |
|  |  |  | **BIPOC** |  |  |  |  |
|  | No | | Yes | | Total | |  |
|  | n | % | n | % | n | % | Pr > Chisq |
| Acceptable | 207 | 48.0 | 118 | 50.9 | 325 | 43.7 |  |
| Potentially acceptable | 141 | 32.7 | 91 | 39.2 | 232 | 31.2 |  |
| Not acceptable | 83 | 19.3 | 23 | 9.9 | 106 | 14.3 |  |
| Total | 431 | 100.0 | 232 | 100.0 | 663 | 89.2 | 0.006 |

Differences in acceptability of using family data by BIPOC status
Question: For you personally, how acceptable is it to use family information for suicide risk prediction?

Table S4. Acceptability of Linking Financial Information by Experience with Negative Life Event

|  | | | | | | |
| --- | --- | --- | --- | --- | --- | --- |
| Among those with experience with suicidality (self or someone you know) | | | | | |  |
|  | Willing | | Private | | Total | |
|  | n | % | n | % | n | % |
| Acceptable | 68 | 28.5 | 4 | 4.2 | 72 | 21.6 |
| Potentially acceptable | 131 | 54.8 | 34 | 35.8 | 165 | 49.4 |
| Not acceptable | 40 | 16.7 | 57 | 60 | 97 | 29.0 |
| Total | 239 | 71.6 | 95 | 28.4 | 334 | 100.0 |
| Pr > Chisq <0.001 |  |  |  |  |  |  |
|  |  |  |  |  |  |  |
| Among those with experience with mental health issues (self or someone you know) | | | | | | |
|  | Willing | | Private | | Total | |
|  | n | % | n | % | n | % |
| Acceptable | 106 | 27.5 | 9 | 6.0 | 115 | 21.5 |
| Potentially acceptable | 208 | 54 | 54 | 36.2 | 262 | 49.1 |
| Not acceptable | 71 | 18.4 | 86 | 57.7 | 157 | 29.4 |
| Total | 385 | 72.1 | 149 | 27.9 | 534 | 100.0 |
| Pr > Chisq <0.001 |  |  |  |  |  |  |
|  |  |  |  |  |  |  |
| Among those with experience with serious financial setbacks (self or someone you know) | | | | | | |
|  | Willing | | Private | | Total | |
|  | n | % | n | % | n | % |
| Acceptable | 66 | 27.4 | 5 | 5.4 | 71 | 21.3 |
| Potentially acceptable | 138 | 57.3 | 31 | 33.3 | 169 | 50.6 |
| Not acceptable | 37 | 15.3 | 57 | 61.3 | 94 | 28.1 |
| Total | 241 | 72.2 | 93 | 27.8 | 334 | 100.0 |
| Pr > Chisq <0.001 |  |  |  |  |  |  |

Table S5. Acceptability of Linking Legal Information by Experience with Negative Life Event

|  | | | | | | |
| --- | --- | --- | --- | --- | --- | --- |
| Among those with experience with suicidality (self or someone you know) | | | | | |  |
|  | Willing | | Private | | Total | |
|  | n | % | n | % | n | % |
| Acceptable | 108 | 45.2 | 20 | 21.1 | 128 | 38.3 |
| Potentially acceptable | 106 | 44.4 | 31 | 32.6 | 137 | 41.0 |
| Not acceptable | 25 | 10.5 | 44 | 46.3 | 69 | 20.7 |
| Total | 239 | 71.6 | 95 | 28.4 | 334 | 100.0 |
| Pr > Chisq <0.001 |  |  |  |  |  |  |
|  |  |  |  |  |  |  |
| Among those with experience with mental health issues (self or someone you know) | | | | | | |
|  | Willing | | Private | | Total | |
|  | n | % | n | % | n | % |
| Acceptable | 165 | 42.9 | 32 | 21.5 | 197 | 36.9 |
| Potentially acceptable | 173 | 44.9 | 51 | 34.2 | 224 | 41.9 |
| Not acceptable | 47 | 12.2 | 66 | 44.3 | 113 | 21.2 |
| Total | 385 | 72.1 | 149 | 27.9 | 534 | 100.0 |
| Pr > Chisq <0.001 |  |  |  |  |  |  |
|  |  |  |  |  |  |  |
| Among those with experience with serious legal issues (self or someone you know) | | | | | | |
|  | Willing | | Private | | Total | |
|  | n | % | n | % | n | % |
| Acceptable | 52 | 41.6 | 10 | 15.4 | 62 | 32.6 |
| Potentially acceptable | 58 | 46.4 | 27 | 41.5 | 85 | 44.7 |
| Not acceptable | 15 | 12 | 28 | 43.1 | 43 | 22.6 |
| Total | 125 | 65.8 | 65 | 34.2 | 190 | 100.0 |
| Pr > Chisq <0.001 |  |  |  |  |  |  |

Table S6. Acceptability of Linking Family Information by Experience with Negative Life Event

|  | | | | | | |
| --- | --- | --- | --- | --- | --- | --- |
| Among those with experience with suicidality (self or someone you know) | | | | | |  |
|  | Willing | | Private | | Total | |
|  | n | % | n | % | n | % |
| Acceptable | 147 | 62.3 | 21 | 22.6 | 168 | 50.3 |
| Potentially acceptable | 76 | 32.2 | 35 | 37.6 | 111 | 33.2 |
| Not acceptable | 13 | 5.5 | 37 | 39.8 | 50 | 15.0 |
| Missing |  |  |  |  | 5 | 1.5 |
| Total | 236 | 70.7 | 93 | 27.8 | 334 | 100.0 |
| Pr > Chisq <0.001 |  |  |  |  |  |  |
|  |  |  |  |  |  |  |
| Among those with experience with mental health issues (self or someone you know) | | | | | | |
|  | Willing | | Private | | Total | |
|  | n | % | n | % | n | % |
| Acceptable | 232 | 60.9 | 38 | 26.0 | 270 | 50.6 |
| Potentially acceptable | 124 | 32.6 | 55 | 37.7 | 179 | 33.5 |
| Not acceptable | 25 | 6.6 | 53 | 36.3 | 78 | 14.6 |
| Missing |  |  |  |  | 7 | 1.3 |
| Total | 381 | 71.3 | 146 | 27.3 | 534 | 100.0 |
| Pr > Chisq <0.001 |  |  |  |  |  |  |
|  |  |  |  |  |  |  |
| Among those with experience with family court (self or someone you know) | | | | | |  |
|  | Willing | | Private | | Total | |
|  | n | % | n | % | n | % |
| Acceptable | 73 | 56.6 | 13 | 22.4 | 86 | 45.5 |
| Potentially acceptable | 46 | 35.7 | 26 | 44.8 | 72 | 38.1 |
| Not acceptable | 10 | 7.8 | 19 | 32.8 | 29 | 15.3 |
| Missing |  |  |  |  | 2 | 1.1 |
| Total | 129 | 68.3 | 58 | 30.7 | 189 | 100.0 |
| Pr > Chisq <0.001 |  |  |  |  |  |  |

Table S7. Willingness to link data by administrative race

| Willingness by Race recorded in administrative records (rather than self-report) | | | | | |  |
| --- | --- | --- | --- | --- | --- | --- |
|  | Willing | | Private | | Total | |
|  | n | % | n | % | n | % |
| American Indian, Alaska Native | 2 | 0.4 | 1 | 0.4 | 3 | 0.4 |
| Asian | 86 | 17.7 | 33 | 12.8 | 119 | 16.0 |
| Black | 32 | 6.6 | 15 | 5.8 | 47 | 6.3 |
| Hawaiian, Pacific Islander | 9 | 1.9 | 1 | 0.4 | 10 | 1.3 |
| More than one race | 32 | 6.6 | 18 | 7.0 | 50 | 6.7 |
| Unknown | 25 | 5.2 | 9 | 3.5 | 34 | 4.6 |
| White | 299 | 61.6 | 181 | 70.2 | 480 | 64.6 |
| Total | 485 | 65.3 | 258 | 34.7 | 743 | 100.0 |
| Pr > Chisq = 0.21 |  |  |  |  |  |  |
|  |  |  |  |  |  |  |
| Willingness by Ethnicity recorded in administrative records (rather than self-report) | | | | | | |
|  | Willing | | Private | | Total | |
|  | n | % | n | % | n | % |
| Hispanic or Latino | 46 | 69.7 | 20 | 30.3 | 66 | 8.9 |
| Not Hispanic or Latino | 368 | 64.8 | 200 | 35.2 | 568 | 76.4 |
| Unknown | 71 | 65.1 | 38 | 34.9 | 109 | 14.7 |
| Total | 485 | 65.3 | 258 | 34.7 | 743 | 100.0 |
| Pr > Chisq = 0.73 |  |  |  |  |  |  |

Table S8. Willingness by presence of any mental health diagnosis code in administrative records rather than self-reported experience by self or others

| Mental Health Diagnosis | Willing | | Private | | Total | |
| --- | --- | --- | --- | --- | --- | --- |
|  | n | % | n | % | n | % |
| No | 211 | 62.6 | 126 | 37.4 | 337 | 45.4 |
| Yes | 274 | 67.5 | 132 | 32.5 | 406 | 54.6 |
| Total | 485 | 65.3 | 258 | 34.7 | 743 | 100.0 |
| Pr > chisq = 0.165 |  |  |  |  |  |  |

Table S9. Acceptability by information type and administrative race

| Acceptability of Linking Financial Information by Race Recorded in Administrative Records | | | | | | | | |
| --- | --- | --- | --- | --- | --- | --- | --- | --- |
|  | Acceptable | | Potentially acceptable | | Not acceptable | | Total | |
|  | n | % | n | % | n | % | n | % |
| American Indian, Alaska Native | 0 | 0.0 | 2 | 66.7 | 1 | 33.3 | 3 | 0.40 |
| Asian | 28 | 23.7 | 62 | 52.5 | 28 | 23.7 | 118 | 15.88 |
| Black | 9 | 19.2 | 20 | 42.6 | 18 | 38.3 | 47 | 6.33 |
| Hawaiian, Pacific Islander | 0 | 0.0 | 7 | 70.0 | 3 | 30.0 | 10 | 1.35 |
| More than one race | 11 | 22.0 | 24 | 48.0 | 15 | 30.0 | 50 | 6.73 |
| Unknown | 8 | 23.5 | 15 | 44.1 | 11 | 32.4 | 34 | 4.58 |
| White | 97 | 20.3 | 218 | 45.5 | 164 | 34.2 | 479 | 64.47 |
| Missing |  |  |  |  |  |  | 2 | 0.27 |
| Total | 153 | 20.6 | 348 | 46.8 | 240 | 32.3 | 743 | 100.00 |

| Acceptability of Linking Legal Information by Race Recorded in Administrative Records | | | | | | | | |
| --- | --- | --- | --- | --- | --- | --- | --- | --- |
|  | Acceptable | | Potentially acceptable | | Not acceptable | | Total | |
|  | n | % | n | % | n | % | n | % |
| American Indian, Alaska Native | 0 | 0.0 | 3 | 100.0 | 0 | 0.0 | 3 | 0.40 |
| Asian | 51 | 43.6 | 49 | 41.9 | 17 | 14.5 | 117 | 15.75 |
| Black | 12 | 25.5 | 22 | 46.8 | 13 | 27.7 | 47 | 6.33 |
| Hawaiian, Pacific Islander | 2 | 20.0 | 7 | 70.0 | 1 | 10.0 | 10 | 1.35 |
| More than one race | 21 | 42.0 | 20 | 40.0 | 9 | 18.0 | 50 | 6.73 |
| Unknown | 8 | 23.5 | 18 | 52.9 | 8 | 23.5 | 34 | 4.58 |
| White | 165 | 34.4 | 191 | 39.9 | 123 | 25.7 | 479 | 64.47 |
| Missing |  |  |  |  |  |  | 3 | 0.40 |
| Total | 259 | 34.9 | 310 | 41.7 | 171 | 23.0 | 743 | 100.00 |

| Acceptability of Linking Family Information by Race Recorded in Administrative Records | | | | | | | | |
| --- | --- | --- | --- | --- | --- | --- | --- | --- |
|  | Acceptable | | Potentially acceptable | | Not acceptable | | Total | |
|  | n | % | n | % | n | % | n | % |
| American Indian, Alaska Native | 0 | 0.0 | 3 | 100.0 | 0 | 0.0 | 3 | 0.40 |
| Asian | 61 | 53.5 | 44 | 38.6 | 9 | 7.9 | 114 | 15.34 |
| Black | 24 | 52.2 | 16 | 34.8 | 6 | 13.0 | 46 | 6.19 |
| Hawaiian, Pacific Islander | 4 | 40.0 | 5 | 50.0 | 1 | 10.0 | 10 | 1.35 |
| More than one race | 24 | 48.0 | 19 | 38.0 | 7 | 14.0 | 50 | 6.73 |
| Unknown | 13 | 39.4 | 15 | 45.5 | 5 | 15.2 | 33 | 4.44 |
| White | 226 | 47.8 | 152 | 32.1 | 95 | 20.1 | 473 | 63.66 |
| Missing |  |  |  |  |  |  | 14 | 1.88 |
| Total | 352 | 47.4 | 254 | 34.2 | 123 | 16.6 | 743 | 100.00 |
|  |  |  |  |  |  |  |  |  |

Table S10. Experiences with completing the DCE choice tasks

| The questions were easy to understand | | | | | | |
| --- | --- | --- | --- | --- | --- | --- |
|  | Willing | | Private | | Total | |
|  | n | % | n | % | n | % |
| Strongly Agree | 67 | 13.9 | 31 | 16.0 | 98 | 13.2 |
| Agree | 227 | 47.0 | 80 | 41.2 | 307 | 41.3 |
| Neither | 78 | 16.2 | 21 | 10.8 | 99 | 13.3 |
| Disagree | 89 | 18.4 | 51 | 26.3 | 140 | 18.8 |
| Strongly Disagree | 22 | 4.6 | 11 | 5.7 | 33 | 4.4 |
| Missing |  |  |  |  | 66 | 8.9 |
| Total | 483 | 65.01 | 194 | 26.1 | 743 | 100.0 |
| p = 0.072 |  |  |  |  |  |  |
|  |  |  |  |  |  |  |
| The questions were easy to answer | | | | | | |
|  | Willing | | Private | | Total | |
|  | n | % | n | % | n | % |
| Strongly Agree | 65 | 13.5 | 40 | 20.6 | 105 | 14.1 |
| Agree | 208 | 43.1 | 75 | 38.7 | 283 | 38.1 |
| Neither | 94 | 19.5 | 32 | 16.5 | 126 | 17.0 |
| Disagree | 100 | 20.7 | 43 | 22.2 | 143 | 19.2 |
| Strongly Disagree | 16 | 3.3 | 4 | 2.1 | 20 | 2.7 |
| Missing |  |  |  |  | 66 | 8.9 |
| Total | 483 | 65.0 | 194 | 26.1 | 743 | 100.0 |
| p = 0.145 |  |  |  |  |  |  |
|  |  |  |  |  |  |  |
| My answers show my real preferences | | | | | | |
|  | Willing | | Private | | Total | |
|  | n | % | n | % | n | % |
| Strongly Agree | 109 | 22.5 | 67 | 34.5 | 176 | 23.7 |
| Agree | 248 | 51.2 | 92 | 47.4 | 340 | 45.8 |
| Neither | 92 | 19.0 | 25 | 12.9 | 117 | 15.7 |
| Disagree | 28 | 5.8 | 8 | 4.1 | 36 | 4.8 |
| Strongly Disagree | 7 | 1.5 | 2 | 1.0 | 9 | 1.2 |
| Missing |  |  |  |  | 65 | 8.7 |
| Total | 484 | 65.1 | 194 | 26.1 | 743 | 100.0 |
| p = 0.017 |  |  |  |  |  |  |
|  |  |  |  |  |  |  |
| The questions were relevant to me | | | | | | |
|  | Willing | | Private | | Total | |
|  | n | % | n | % | n | % |
| Strongly Agree | 44 | 9.1 | 30 | 15.5 | 74 | 10.0 |
| Agree | 189 | 39.1 | 57 | 29.4 | 246 | 33.1 |
| Neither | 140 | 28.9 | 60 | 30.1 | 200 | 26.9 |
| Disagree | 85 | 17.6 | 31 | 16.0 | 116 | 15.6 |
| Strongly Disagree | 26 | 5.4 | 16 | 8.3 | 42 | 5.7 |
| Missing |  |  |  |  | 65 | 8.7 |
| Total | 484 |  | 194 |  | 743 | 100.0 |
| p = 0.027 |  |  |  |  |  |  |

Table S11. Questions were easy to understand by NLE

| Easy to understand by Experience with Mental Health Issues | | | | | | |
| --- | --- | --- | --- | --- | --- | --- |
|  | No | | Yes | | Total | |
|  | n | % | n | % | n | % |
| Strongly Agree | 19 | 13.67 | 79 | 14.8 | 98 | 13.2 |
| Agree | 63 | 45.32 | 242 | 45.4 | 305 | 41.0 |
| Neither | 25 | 17.99 | 74 | 13.9 | 99 | 13.3 |
| Disagree | 26 | 18.71 | 112 | 21.0 | 138 | 18.6 |
| Strongly Disagree | 6 | 4.32 | 26 | 4.9 | 32 | 4.3 |
| Missing |  |  |  |  | 71 | 9.6 |
| Total | 139 | 18.7 | 533 | 71.7 | 743 | 100.0 |
| p = 0.787 |  |  |  |  |  |  |
|  |  |  |  |  |  |  |
| Easy to understand by Experience with Financial Issues | | | | | | |
|  | No | | Yes | | Total | |
|  | n | % | n | % | n | % |
| Strongly Agree | 42 | 12.7 | 56 | 16.8 | 98 | 13.2 |
| Agree | 149 | 44.9 | 150 | 45.0 | 299 | 40.2 |
| Neither | 56 | 16.9 | 43 | 12.9 | 99 | 13.3 |
| Disagree | 68 | 20.5 | 69 | 20.7 | 137 | 18.4 |
| Strongly Disagree | 17 | 5.1 | 15 | 4.5 | 32 | 4.3 |
| Missing |  |  |  |  | 78 | 10.5 |
| Total | 332 | 44.7 | 333 | 44.8 | 743 | 100.0 |
| p = 0.428 |  |  |  |  |  |  |
|  |  |  |  |  |  |  |
| Easy to understand by Experience with Legal Issues | | | | | | |
|  | No | | Yes | | Total | |
|  | n | % | n | % | n | % |
| Strongly Agree | 62 | 13.03 | 35 | 18.5 | 97 | 13.1 |
| Agree | 214 | 44.96 | 87 | 46.0 | 301 | 40.5 |
| Neither | 72 | 15.13 | 27 | 14.3 | 99 | 13.3 |
| Disagree | 103 | 21.64 | 32 | 16.9 | 135 | 18.2 |
| Strongly Disagree | 25 | 5.25 | 8 | 4.2 | 33 | 4.4 |
| Missing |  |  |  |  | 78 | 10.5 |
| Total | 476 | 64.1 | 189 | 25.4 | 743 | 100.0 |
| p = 0.428 |  |  |  |  |  |  |
|  |  |  |  |  |  |  |
| Easy to understand by Experience with Family Issues | | | | | | |
|  | No | | Yes | | Total | |
|  | n | % | n | % | n | % |
| Strongly Agree | 65 | 13.54 | 33 | 17.6 | 98 | 13.2 |
| Agree | 217 | 45.21 | 86 | 45.7 | 303 | 40.8 |
| Neither | 72 | 15.00 | 27 | 14.4 | 99 | 13.3 |
| Disagree | 105 | 21.88 | 31 | 16.5 | 136 | 18.3 |
| Strongly Disagree | 21 | 4.38 | 11 | 5.9 | 32 | 4.3 |
| Missing |  |  |  |  | 75 | 10.1 |
| Total | 480 | 64.6 | 188 | 25.3 | 743 | 100.0 |
| p = 0.397 |  |  |  |  |  |  |

Table S12. Questions were easy to answer by NLE

| Easy to answer by Experience with Mental Health Issues | | | | |  |  |
| --- | --- | --- | --- | --- | --- | --- |
|  | No | | Yes | | Total | |
|  | n | % | n | % | n | % |
| Strongly Agree | 20 | 14.39 | 85 | 15.9 | 105 | 14.1 |
| Agree | 52 | 37.41 | 229 | 43.0 | 281 | 37.8 |
| Neither | 34 | 24.46 | 91 | 17.1 | 125 | 16.8 |
| Disagree | 27 | 19.42 | 114 | 21.4 | 141 | 19.0 |
| Strongly Disagree | 6 | 4.32 | 14 | 2.6 | 20 | 2.7 |
| Missing |  |  |  |  | 71 | 9.6 |
| Total | 139 | 18.7 | 533 | 71.7 | 743 | 100.0 |
| p = 0.242 |  |  |  |  |  |  |
|  |  |  |  |  |  |  |
| Easy to answer by Experience with Financial Issues | | | |  |  |  |
|  | No | | Yes | | Total | |
|  | n | % | n | % | n | % |
| Strongly Agree | 45 | 13.55 | 59 | 17.7 | 104 | 14.0 |
| Agree | 141 | 42.47 | 136 | 40.8 | 277 | 37.3 |
| Neither | 64 | 19.28 | 61 | 18.3 | 125 | 16.8 |
| Disagree | 68 | 20.48 | 71 | 21.3 | 139 | 18.7 |
| Strongly Disagree | 14 | 4.22 | 6 | 1.8 | 20 | 2.7 |
| Missing |  |  |  |  | 78 | 10.5 |
| Total | 332 | 44.7 | 333 | 44.8 | 743 | 100.0 |
| p = 0.257 |  |  |  |  |  |  |
|  |  |  |  |  |  |  |
| Easy to answer by Experience with Legal Issues | | | |  |  |  |
|  | No | | Yes | | Total | |
|  | n | % | n | % | n | % |
| Strongly Agree | 72 | 15.13 | 32 | 16.9 | 104 | 14.0 |
| Agree | 193 | 40.55 | 85 | 45.0 | 278 | 37.4 |
| Neither | 94 | 19.75 | 30 | 15.9 | 124 | 16.7 |
| Disagree | 103 | 21.64 | 36 | 19.0 | 139 | 18.7 |
| Strongly Disagree | 14 | 2.94 | 6 | 3.2 | 20 | 2.7 |
| Missing |  |  |  |  | 78 | 10.5 |
| Total | 476 | 64.1 | 189 | 25.4 | 743 | 100.0 |
| p = 0.651 |  |  |  |  |  |  |
|  |  |  |  |  |  |  |
| Easy to answer by Experience with Family Issues | | | |  |  |  |
|  | No | | Yes | | Total | |
|  | n | % | n | % | n | % |
| Strongly Agree | 76 | 15.97 | 29 | 15.3 | 105 | 14.1 |
| Agree | 192 | 40.34 | 89 | 47.1 | 281 | 37.8 |
| Neither | 97 | 20.38 | 27 | 14.3 | 124 | 16.7 |
| Disagree | 102 | 21.43 | 38 | 20.1 | 140 | 18.8 |
| Strongly Disagree | 13 | 2.73 | 5 | 2.6 | 18 | 2.4 |
| Missing |  |  |  |  | 75 | 10.1 |
| Total | 480 | 64.6 | 188 | 25.3 | 743 | 100.0 |
| p = 0.367 |  |  |  |  |  |  |

Table S13. Answers reflect real preferences by NLE

| My Answers show my real preferences by Experience with Mental Health Issues | | | | | | |
| --- | --- | --- | --- | --- | --- | --- |
|  | No | | Yes | | Total | |
|  | n | % | n | % | n | % |
| Strongly Agree | 37 | 26.43 | 137 | 25.7 | 174 | 23.4 |
| Agree | 69 | 49.29 | 269 | 50.5 | 338 | 45.5 |
| Neither | 30 | 21.43 | 86 | 16.1 | 116 | 15.6 |
| Disagree | 2 | 1.43 | 34 | 6.4 | 36 | 4.8 |
| Strongly Disagree | 2 | 1.43 | 7 | 1.3 | 9 | 1.2 |
| Missing |  |  |  |  | 70 | 9.4 |
| Total | 140 | 18.8 | 533 | 71.7 | 743 | 100.0 |
| p = 0.139 |  |  |  |  |  |  |
|  |  |  |  |  |  |  |
| My Answers show my real preferences by Experience with Financial Issues | | | | | |  |
|  | No | | Yes | | Total | |
|  | n | % | n | % | n | % |
| Strongly Agree | 85 | 25.53 | 87 | 26.1 | 172 | 23.1 |
| Agree | 168 | 50.45 | 166 | 49.8 | 334 | 45.0 |
| Neither | 62 | 18.62 | 54 | 16.2 | 116 | 15.6 |
| Disagree | 14 | 4.20 | 21 | 6.3 | 35 | 4.7 |
| Strongly Disagree | 4 | 1.20 | 5 | 1.5 | 9 | 1.2 |
| Missing |  |  |  |  | 77 | 10.4 |
| Total | 333 | 44.8 | 333 | 44.8 | 743 | 100.0 |
| p = 0.718 |  |  |  |  |  |  |
|  |  |  |  |  |  |  |
| My Answers show my real preferences by Experience with Legal Issues | | | | | |  |
|  | No | | Yes | | Total | |
|  | n | % | n | % | n | % |
| Strongly Agree | 123 | 25.79 | 49 | 25.9 | 172 | 23.1 |
| Agree | 239 | 50.10 | 95 | 50.3 | 334 | 45.0 |
| Neither | 85 | 17.82 | 30 | 15.9 | 115 | 15.5 |
| Disagree | 23 | 4.82 | 13 | 6.9 | 36 | 4.8 |
| Strongly Disagree | 7 | 1.47 | 2 | 1.1 | 9 | 1.2 |
| Missing |  |  |  |  | 77 | 10.4 |
| Total | 477 | 64.2 | 189 | 25.4 | 743 | 100.0 |
| p = 0.822 |  |  |  |  |  |  |
|  |  |  |  |  |  |  |
| My Answers show my real preferences by Experience with Family Issues | | | | | |  |
|  | No | | Yes | | Total | |
|  | n | % | n | % | n | % |
| Strongly Agree | 131 | 27.23 | 44 | 23.4 | 175 | 23.6 |
| Agree | 231 | 48.02 | 104 | 55.3 | 335 | 45.1 |
| Neither | 88 | 18.30 | 26 | 13.8 | 114 | 15.3 |
| Disagree | 24 | 4.99 | 12 | 6.4 | 36 | 4.8 |
| Strongly Disagree | 7 | 1.46 | 2 | 1.1 | 9 | 1.2 |
| Missing |  |  |  |  | 74 | 10.0 |
| Total | 481 | 64.7 | 188 | 25.3 | 743 | 100.0 |
| p = 0.352 |  |  |  |  |  |  |

Table S14. Relevance of Questions by NLE

| The Questions were Relevant to me by Experience with Mental Health Issues | | | | | | |
| --- | --- | --- | --- | --- | --- | --- |
|  | No | | Yes | | Total | |
|  | n | % | n | % | n | % |
| Strongly Agree | 10 | 7.14 | 63 | 11.82 | 73 | 9.8 |
| Agree | 36 | 25.71 | 210 | 39.40 | 246 | 33.1 |
| Neither | 45 | 32.14 | 153 | 28.71 | 198 | 26.6 |
| Disagree | 31 | 22.14 | 84 | 15.76 | 115 | 15.5 |
| Strongly Disagree | 18 | 12.86 | 23 | 4.32 | 41 | 5.5 |
| Missing |  |  |  |  | 70 | 9.4 |
| Total | 140 | 18.8 | 533 | 71.7 | 743 | 100.0 |
| p < 0.001 |  |  |  |  |  |  |
|  |  |  |  |  |  |  |
| The Questions were Relevant to me by Experience with Financial Issues | | | | | | |
|  | No | | Yes | | Total | |
|  | n | % | n | % | n | % |
| Strongly Agree | 25 | 7.53 | 48 | 14.37 | 73 | 9.8 |
| Agree | 113 | 34.04 | 129 | 38.62 | 242 | 32.6 |
| Neither | 100 | 30.12 | 97 | 29.04 | 197 | 26.5 |
| Disagree | 68 | 20.48 | 45 | 13.47 | 113 | 15.2 |
| Strongly Disagree | 26 | 7.83 | 15 | 4.49 | 41 | 5.5 |
| Missing |  |  |  |  | 77 | 10.4 |
| Total | 332 | 44.7 | 334 | 45.0 | 743 | 100.0 |
| p = 0.003 |  |  |  |  |  |  |
|  |  |  |  |  |  |  |
| The Questions were relevant to me by Experience with Legal Issues | | | | | | |
|  | No | | Yes | | Total | |
|  | n | % | n | % | n | % |
| Strongly Agree | 48 | 10.08 | 24 | 12.63 | 72 | 9.7 |
| Agree | 173 | 36.34 | 69 | 36.32 | 242 | 32.6 |
| Neither | 137 | 28.78 | 60 | 31.58 | 197 | 26.5 |
| Disagree | 90 | 18.91 | 25 | 13.16 | 115 | 15.5 |
| Strongly Disagree | 28 | 5.88 | 12 | 6.32 | 40 | 5.4 |
| Missing |  |  |  |  | 77 | 10.4 |
| Total | 476 | 64.1 | 190 | 25.6 | 743 | 100.0 |
| p = 0.431 |  |  |  |  |  |  |
|  |  |  |  |  |  |  |
| The Questions were relevant to me by Experience with Family Issues | | | | | | |
|  | No | | Yes | | Total | |
|  | n | % | n | % | n | % |
| Strongly Agree | 49 | 10.21 | 24 | 12.7 | 73 | 9.8 |
| Agree | 160 | 33.33 | 84 | 44.4 | 244 | 32.8 |
| Neither | 146 | 30.42 | 52 | 27.5 | 198 | 26.6 |
| Disagree | 92 | 19.17 | 22 | 11.6 | 114 | 15.3 |
| Strongly Disagree | 33 | 6.88 | 7 | 3.7 | 40 | 5.4 |
| Missing |  |  |  |  | 74 | 10.0 |
| Total | 480 | 64.6 | 189 | 25.4 | 743 | 100.0 |
| p = 0.0138 |  |  |  |  |  |  |
